# Diagnosis and Management of Polyorchidism: A Case Report and Literature Review

**DOI:** 10.1155/2023/1620276

**Published:** 2023-06-22

**Authors:** Anthony Kanbar, Charbel Dabal, Joey El Khoury, Rami Halabi, Serge Assaf, Anthony Mina, Sabine Breidi, Maher Abdessater, Raghid El Khoury

**Affiliations:** ^1^School of Medicine and Medical Sciences, Holy Spirit University of Kaslik (USEK), P.O. Box 446, Jounieh, Lebanon; ^2^Urology Department, Notre Dame des Secours University Hospital Center (CHUNDS), Byblos City, Lebanon

## Abstract

Polyorchidism, or supernumerary testis, is a rare congenital abnormality of the genitourinary system. In this paper, we present the case of triorchidism in a seven-year-old asymptomatic child with a suspect left scrotal mass detected on routine physical examination. Imaging studies revealed a third testicle in the left hemiscrotum, with comparable dimensions, signal intensity on MRI, and Doppler flow on ultrasound with the ipsilateral testis. We also discuss the clinical presentations, classifications, and current diagnostic and therapeutic strategies of this condition.

## 1. Introduction

Polyorchidism, also known as supernumerary testis, is a rare congenital abnormality of the genitourinary system characterized by the presence of more than two testicles. It is a sporadic phenomenon whose etiology remains unclear [1], with less than 250 cases documented in the medical literature [2, 3]. Polyorchidism is believed to result from the accidental division of the germinal ridge before the eighth week of embryologic development [3, 4]. The supernumerary testis may share epididymis with the adjacent testis or have its own. In the majority of cases [2], it is connected to a vas deferens and is classified as type A, as per Balawender et al.'s [3] classification; otherwise, it is classified as type B. Polyorchidism is associated with inguinal hernia, cryptorchidism, hydrocele, testicular torsion, and an increased risk of testicular cancer. Most patients are asymptomatic, and the diagnosis is usually incidental during the evaluation for other symptoms [5]. Although imaging techniques have facilitated diagnosis, the management of polyorchidism remains controversial due to the increased risk of malignancy. In this case report, we present a case of triorchidism in an asymptomatic child and discuss the current diagnostic and therapeutic strategies.

## 2. Case Presentation

Our urology clinic received a referral from the patient's pediatrician for a left scrotal asymptomatic mass in a 7-year-old male patient, detected during a routine physical examination. The patient's medical and surgical history was unremarkable, and he was observed to be developing normally in all other aspects.

Upon physical examination, the patient presented a visible swelling on the left hemiscrotum. On the right side, the testicle and epididymis had normal consistencies and volumes, the vas deferens was palpable, and no inguinal hernia or varicocele veins were identified. However, the patient had a grade IV varicocele on the left side, accompanied by atrophic testicle and epididymis. Additionally, the left vas deferens was palpable and more prominent than its right-sided counterpart. A medial para testicular mass was palpated on the left side, exhibiting comparable volume and consistency with the atrophic testis. The levels of serum tumor markers for testicular cancer, including alpha-fetoprotein, human chorionic gonadotropin, and lactate dehydrogenase, were found to be within the normal ranges.

**Figure 1:**
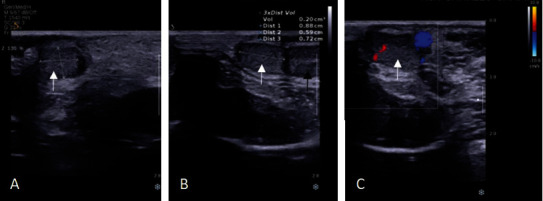
Ultrasonography in a seven-year-old boy showing a slightly echoic oval-shaped structure (white arrow), compared to the left testicular parenchyma (black arrow), located between the left epididymis and the left testicle, and measuring 0.9 × 0.6 × 0.7 cm. It is completely separated from the left testicle and shows mild intralesional Doppler flow (C) with a slight posterior reinforcement, compatible with a supernumerary testis.

**Figure 2:**
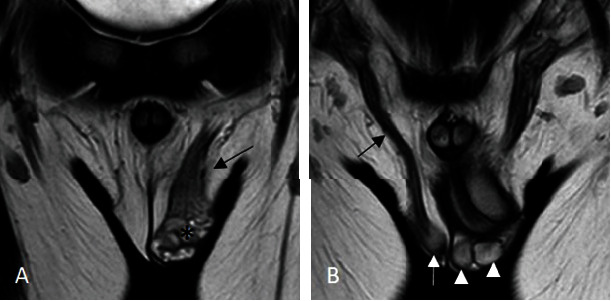
Magnetic resonance imaging in a seven-year-old boy diagnosed with polyorchidism. T2-weighted images in coronal sections, showing normally positioned right testicle (white arrow), and two testicles at the left side (white arrowheads) with homogenous and comparable hypersignal. Varicose scrotal veins are noted on the left side (star). A single epididymis and cord drained both left structures that was thicker (black arrow in (A)) and wider than on the right side (black arrow in (B)).

The ultrasound examination revealed a somewhat echogenic, oval-shaped entity measuring 0.9 × 0.6 × 0.7 cm, positioned between the left epididymis and the left testicle. The mass was distinct from the left testis and exhibited slight intralesional Doppler flow with posterior reinforcement (Figure 1). To enhance our assessment, a testicular MRI was conducted (Figure 2). The imaging revealed a normal testicle measuring 15.2 × 12 × 10.2 mm on the right side. However, two oval-shaped structures were detected on the left side with comparable dimensions and signal intensity (hypointense on diffusion, isosignal on T1, and hypersignal on T2, compared to the right testis). These structures measured 9.5 × 7.5 × 7.5 mm each and were surrounded by varicosal veins. Additionally, only one epididymis was identified on the left side, and a single cord was found to drain both left structures. It is worth noting that the left cord was thicker and wider (10 mm) than the right one (4 mm).

The collective findings were consistent with the presence of a third testicle. The varicocele on the left scrotum was surgically addressed through laparoscopic vein ligation, and the supernumerary testis was conserved. The potential hazards of testicular torsion and subfertility were thoroughly deliberated with the parents, and a comprehensive follow-up plan was outlined.

## 3. Discussion

Supernumerary testis or polyorchidism is a rare congenital anomaly. Since its first description by Lane et al. in 1895, less than 250 cases have been reported in literature [1, 3]. It is often associated with anomalies of the processus vaginalis and carries an increased risk of malignancy and infertility. The anomaly is thought to result from an accidental division of the genital ridge prior to the eighth week of embryological development. While there are numerous embryological theories to explain its pathogenesis, including anomalous appropriation of cells, duplication or division of the urogenital ridge, incomplete degeneration of the mesonephros, and development of peritoneal bands, these theories remain insufficient to explain all aspects of polyorchidism's pathogenesis [5]. Mittal et al. and Leung categorized this anomaly into four types based on embryologic development, as summarized in Table 1 [6, 7].

More recently, Bergholz et al. suggested a new anatomical classification based on the functional taxonomy of polyorchidism to standardize diagnosis and management based on the reproductive potential of the supernumerary testis, as outlined in Table 2 [8].

The supernumerary testis typically shares epididymis and cord with the ipsilateral testis [5]. While three and four testicles are the most common forms, up to five have been described [1]. Most supernumerary testes are situated within the scrotal region, with fewer instances occurring within inguinal and abdominal locations. They are commonly found on the left side due to reported differences in the topographic vascular anatomy and the size of the left testicle compared to the right one. Nevertheless, the scientific literature has also reported right-sided and bilateral polyorchidism cases [5]. Polyorchidism is most commonly diagnosed during adolescence, with a median age of 17. Typically, patients are diagnosed incidentally while undergoing evaluation for other symptoms. Pain is seldom cited as the chief complaint, with only 7% of patients experiencing it. Polyorchidism is associated with inguinal hernia, cryptorchidism, testicular torsion, and hydrocele in 24%, 22%, 15%, and 9% of cases, respectively [5]. White et al.'s meta-analysis reported a prevalence of 1.4% of varicocele among patients with polyorchidism [9]. Altered or absent spermatogenesis was observed in 11 and 26% of patients, respectively, occurring mainly in the undescended supernumerary testis. Neoplasm rates among supernumerary testis varied between 1 and 7% between series, and cryptorchidism appears to be the most crucial risk factor for malignancy in those patients [5].

Polyorchidism can potentially imitate various pathologies such as varicocele, hydrocele, spermatocele, and testicular neoplasms. Therefore, the physical examination may not be sufficient to diagnose polyorchidism. On ultrasonography (US), a supernumerary testis is identifiable as an oval structure with the same echogenicity as the normal testis. Magnetic resonance imaging (MRI) can provide supplementary information if the diagnosis cannot be made using US. The supernumerary testis exhibits identical signal intensities to the normal testis on MRI, including intermediate and high signal intensities in T1 and T2 sequences, respectively. Some authors suggested using serological markers and histologic confirmation when imaging cannot differentiate polyorchidism from other intrascrotal pathologies [2].

The management of polyorchidism remains controversial, as no evidence-based approach is currently available. Over time, management strategies have evolved, with advancements in imaging techniques enabling surveillance to replace surgical interventions (excision, exploration, and biopsy). When deciding on the optimal management option for supernumerary testis, it is crucial to consider the heightened risks of testicular torsion and malignancy [2] while preserving the reproductive potential. In addition, factors such as compliance with surveillance, parental preference, and cosmesis must be considered [6]. Cryptorchidism appears to be the most important risk factor for malignancy in patients with supernumerary testis [10]. The previous practice involved removing the supernumerary testis (usually the smaller one) irrespective of its position. However, more conservative approaches are now followed, thanks to the advances in the radiological characterization of suspect masses. Surgical management has to be considered primarily in type B supernumerary testis that does not contribute to fertility (lacks an out-flow path) and for patients with cryptorchid supernumerary testis due to a heightened risk of malignancy. Patients requiring surgical intervention for associated anomalies such as inguinal hernia and cryptorchidism can benefit from the intraoperative frozen section for histological evaluation, with or without orchiectomy (if there are signs of malignancy) or orchiopexy (to prevent future testicular torsion). In cases where a supernumerary testis is detected by imaging and not associated with any other abnormality requiring surgical intervention, conservative treatment, with watchful waiting and regular follow-up, is appropriate [5]. Some authors suggest annual physical examination, serum tumor marker check, and US for the follow-up, although further studies are necessary to determine the optimal follow-up strategy [2].

Our patient presented with a supernumerary testis in the scrotal region, a feature commonly observed and documented in literature [3]. It shared the epididymis and cord with the ipsilateral testis and fell under the A3 classification (like 16% of reported cases) [8], potentially participating in spermatogenesis despite its reduced size. Since the patient did not exhibit any anomalies that required open surgical repair, and since the supernumerary testis was in the scrotal position, after explaining the risks of torsion, we decided with the parents to keep it and to adopt an active surveillance strategy. We treated only the left varicocele to optimize the patient's chances of fertility. Our management was not altered by the presence of varicocele, due to its low prevalence among patients with polyorchidism (1.4%) compared to the general population (15%), as well as the lack of data on specific associations between the two conditions [3, 9].

## 4. Conclusion

Polyorchidism should be considered a potential diagnosis when assessing scrotal or inguinal masses or pain. It may imitate various pathological conditions, and imaging is helpful for differential diagnosis. Removal of the supernumerary testis must be considered when there is a concern for malignancy (cryptorchid supernumerary testis) and when the testis is not drained. Further research is needed to understand the factors contributing to this disorder and to establish an appropriate management plan.

## Figures and Tables

**Table 1:** Classification of polyorchidism by Mittal et al. and Leung [6, 7].

Type	Characteristics of the supernumerary testis
1	Lacks epididymis or VD
2	Shares a common epididymis and VD with the ipsilateral testicle
3	Has its own epididymis but shares a common VD with the ipsilateral testicle
4	Has its own epididymis and VD

VD: vas deferens.

**Table 2:** Classification of polyorchidism by Bergholz et al. [8].

Type	Drainage status of the supernumerary testis	Subtype	Relation to the epididymis and ipsilateral adjacent testis
A	Drained by a VD	A1	Has its own epididymis and VD
		A2	Has its own epididymis but shares a common VD with adjacent testis
		A3	Shares a common epididymis and VD with adjacent testis

B	Not drained by a VD	B1	Has its own epididymis
		B2	Does not have its own epididymis

VD: vas deferens.

## Data Availability

The radiology images used during the current study are available from the corresponding author upon reasonable request.
